# Optimizing Student Outcomes: A Comparison of Two Teaching Methods for Identifying Vegetal Foreign Bodies in Canine Limbs Using Simulation Models and Ultrasound

**DOI:** 10.1111/vru.70073

**Published:** 2025-08-20

**Authors:** Alexandra Williams, Ebony Schoenfeld, Esther Callcott, Randi Rotne

**Affiliations:** ^1^ School of Agriculture Environmental and Veterinary Science Charles Sturt University Wagga Wagga NSW Australia

**Keywords:** canine interdigital vegetal foreign body, simulation‐based teaching, silicone models, simulation‐based veterinary education, ultrasonography

## Abstract

Musculoskeletal ultrasonography is an underutilized skill within the veterinary industry. It requires practice and repetition to improve confidence and competence. In a clinic, ultrasonography is often used to identify grass seeds in patients; however, its teaching within the veterinary curriculum is limited. This study investigated the use of simulation models using two different teaching methods to improve undergraduate student outcomes in identifying vegetal foreign bodies (VFB). Forelimb models, containing wild oaten grass seeds (*Avena fatua*) in one of three locations, were used as ultrasonographic teaching aids. Fourth‐year undergraduate veterinary science students (*n* = 38) were randomly assigned a limb and taught an ultrasound protocol. The trial group (*n* = 19) received video instructions with a demonstration, whilst the control group (*n* = 19) received written instructions only. All participants completed a pre‐ and post‐ participation survey and were anonymously assessed on their ability to locate the grass seed. Participants provided with both the video and written instructions (trial group) were 1.92 times more likely to identify the VFB within the simulation model when compared with the control group, with 53% identifying the VFB (*p* = .0327). All students agreed they would consider using ultrasonography in the future to identify canine VFBs. The outcomes of this study suggest that the use of simulation models in teaching is beneficial and that visual and verbal communication significantly improved students’ ability to identify VFB compared with written instruction alone in a consequence‐free learning environment.

## 1 Introduction

Vegetal foreign bodies (VFB) are common globally in small animal clinics. They have been reported in Europe, North America, and Australia, where VFB represent 2% of small animal cases seen within the Riverina, New South Wales, Australia [1]. Grass seeds have a unique shape, consisting of a pointed floret with rearward‐facing speared awns, which allow unidirectional migration through the body following penetration of the skin or an orifice [1, 2, 3]. Vegetal foreign bodies can occur anywhere in the body; however, one of the most common locations is the canine distal limb. Specific locations in the distal limb include the interdigital webbing, the metacarpal, metatarsal, and carpal regions [4, 5].

Ultrasound has many benefits for identifying VFB. It can visualize non‐radiopaque foreign bodies, has a high sensitivity for soft tissue, is cost‐effective, and is commonly available in general practice, making it a useful investigative tool [3, 6, 7]. A high‐frequency, linear transducer has been demonstrated to optimize near‐field spatial resolution, which is preferred when identifying VFB [5]. Most VFB studies have utilized a 7.5–14 MHz linear transducer for this purpose [3, 5, 6, 7, 8]. Furthermore, VFB, regardless of species, are described on ultrasound as a hyperechoic spindle shape with two to three linear interfaces enhanced by a hypoechoic region [5, 9, 10].

The most significant limitation in the use of ultrasonography is user error [3, 11]. Ultrasonography is classified as an open, deliberate skill that requires practice, repetition, and familiarity to increase user confidence and diagnostic capability [12, 13]. Traditionally, student learning has involved the use of live patients or cadavers; however, driven by growing concerns for animal welfare, current guidelines require the reduction of animal use where appropriate, refinement of teaching to minimize waste, and/or replacement of animal use when possible (the 3Rs principles) [14, 15]. Consequently, there is increasing evidence to support simulation model use within teaching without compromising student outcomes [12, 15]. Simulation models are physical, interactive tools that offer simplified versions of complex real‐world phenomena and processes. They create a safe yet realistic clinical environment where students can repeatedly practice, enhancing their skills and confidence [12, 13, 16]. Additionally, models also support a student‐centered teaching approach by promoting an inquiry‐based process in a dynamic and interactive setting. Students engage by making decisions and critically reflecting on the outcomes of their actions [16].

Simulators have been used to teach various clinical skills in veterinary education. Simulators have been used for teaching endotracheal intubation [17], dentistry [18], suturing [19], venipuncture [20], and catheterization [21]. Musk et al. [17] demonstrated that practicing endotracheal intubation on a high‐fidelity model before working with live patients improved both student confidence and success rates. Similarly, Lumbis et al. [18] found that 92% of students using low‐fidelity dentistry models performed significantly better than those without access, with all participants acknowledging the value of the ability to practice skills for clinical placements and postgraduation. In ultrasonography, a novel silicone simulation model has recently been developed for imaging VFB in the canine distal limb [22], though its effectiveness as a teaching tool has yet to be demonstrated.

Teaching methods are evolving to align with diverse learning styles, and there is a noticeable shift toward outcome‐based education [14]. This is when simulation‐based training, including the provision of models, becomes important to veterinary curricula. For veterinary students engaged in outcome‐based education, the focus is on acquiring skills where precision is critical for procedures they must perform [16]. Timely feedback and debriefing are essential during simulation‐based teaching [16], whilst incorporating multiple modalities, such as visual, tactile, aural, and verbal elements, has been demonstrated to enhance student learning [23]. Additionally, confidence needs to be considered as this can impact student performance. Previous research has identified a weak correlation between undergraduate students’ confidence and their actual competency [24].

Learning in higher education involves the acquisition of knowledge, the development of critical thinking, and the application of theory to practice [25]. This is especially important where integrating theoretical concepts with clinical skills is essential for producing industry‐ready professionals. Practical training in clinical skills is a well‐established teaching method, with research already revealing that video instruction is more effective than written instruction for both medical professionals and asthma patients when it comes to adhering to protocol‐based procedures [26, 27]. However, to date, there have been no studies validating the use of simulation models for teaching ultrasonography to undergraduate veterinary students, specifically associated with the identification and localization of canine distal limb VFB. This study assessed the use of simulation models using two different teaching methods (video demonstration vs. written instruction) on student outcomes in identifying VFB in canine distal limb simulation models using a predetermined ultrasonographic protocol in a consequence‐free learning environment. It was hypothesized that students would develop improved ultrasonographic identification of grass seeds through the combined use of visual and verbal cues presented in the video demonstration when compared with students who received explicit written instruction only.

## 2 Materials and Methods

### 2.1 Model Design

**FIGURE 1:**
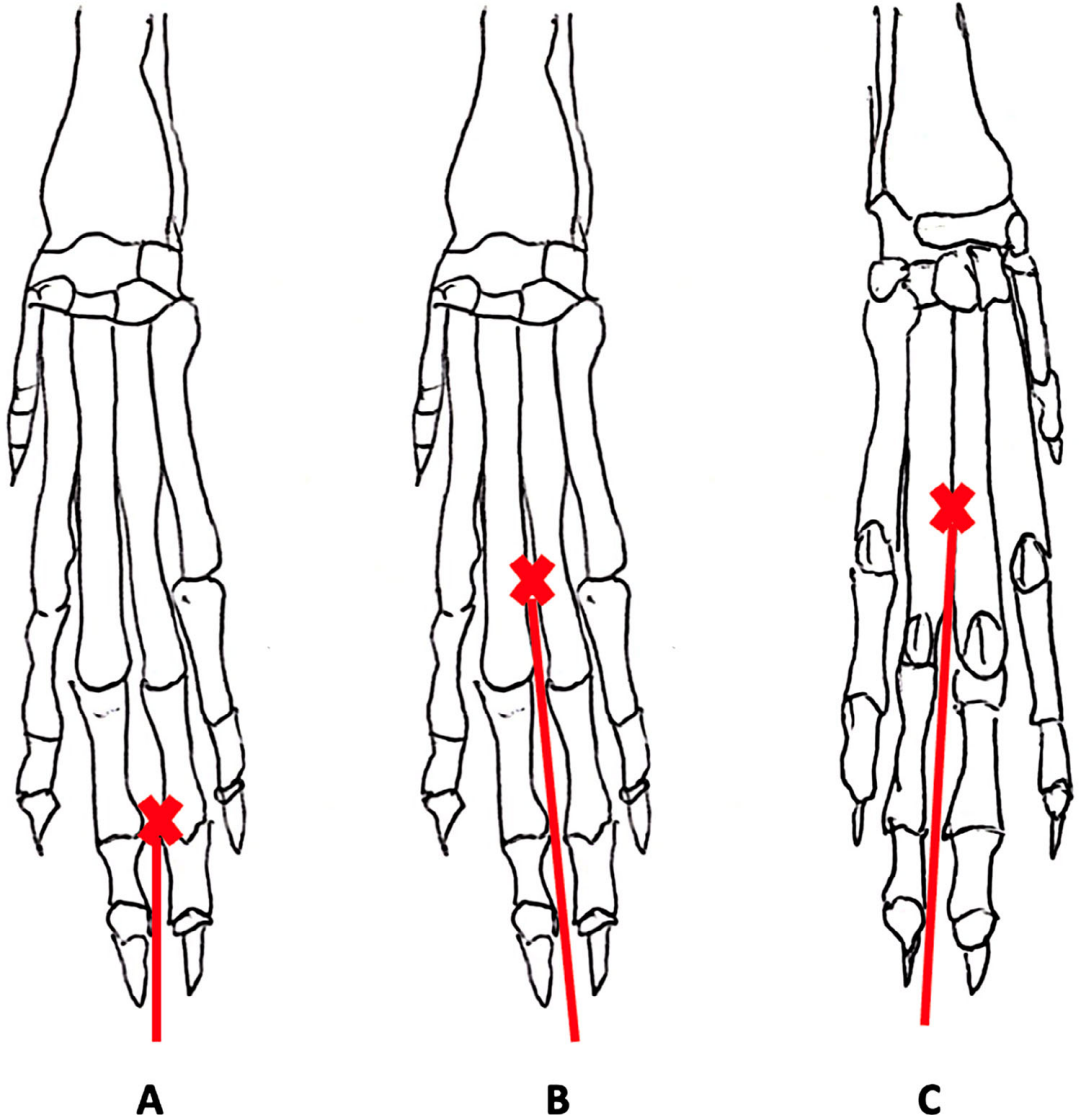
Placement of dried wild oaten grass seed (*Avena fatua*). Grass seeds (**X**) and wooden skewers (red line) were placed into the silicone layers at three locations: (A) interdigital web, (B) dorsal metacarpals, and (C) palmar metacarpals. Image adapted from Schoenfeld et al. [22].

**FIGURE 2:**
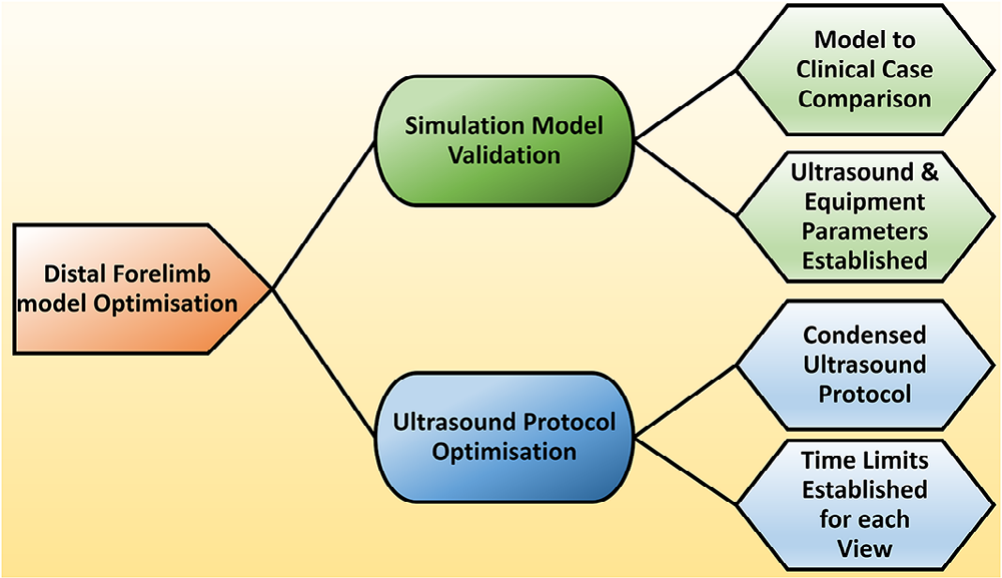
Distal forelimb model testing stages; Stage 1 (Simulation Model Validation) involved assessing the simulation model for likeness to clinical cases and determining the preset ultrasound settings. Stage 2 (Ultrasound Protocol Optimization) involved the testing of the adapted ultrasound protocol. Photos (control) and video (trial) were obtained during Stage 2 testing.

**FIGURE 3:**
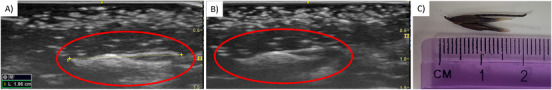
Students were provided with three reference photos (two ultrasound and one physical image) of the dried wild oaten grass seed (*Avena fatua*) prior to ultrasound scanning. (A) First sagittal ultrasound image of a grass seed (red circle) as it appears in the distal forelimb models, (B) second sagittal ultrasound image of a grass seed (red circle) as it appears in the distal forelimb models, and (C) image of a physical grass seed next to a ruler showing approximate seed measurement.

Canine distal forelimb silicone models were developed by Schoenfeld et al. [22] under Charles Sturt University's animal ethics approval number A22305. Dried wild oaten grass seed (Avena fatua) (2 cm length, 2–3 awns) placed in each model were positioned at one of three locations: (1) the interdigital webbing of the 3rd and 4th digits (Figure 1A), (2) the palmar surface of metacarpal 3 and 4 (Figure 1B), or (3) dorsal surface of metacarpal 3 and 4 (Figure 1C). The distal forelimb models (DFM) were color‐coded with tape to identify VFB location and obscured from student view using “6” black Vet Wrap (3 M, Maplewood, Minnesota, USA).

Each DFM was tested for similarity and likeness to live clinical cases. Grass seed visibility [22] and ultrasound settings for student use were determined (Figure 2; Stage 1). Ultrasound settings were determined by an experienced internal medicine consultant with 25 years of experience and an interest in ultrasonography, and aimed at ensuring maximum visibility of the VFB in all DFM. Optimal ultrasonographic factors for the models, including gain, transducer frequency, and dynamic range, were identified and fixed as predetermined settings for the study.

The DFM was next used to test the 13‐step ultrasound protocol (Appendix SA) for teaching capability (Figure 2, Stage 2). A condensed version of the systematic ultrasound protocol to examine the distal limb for the visualization of vegetal foreign bodies (SUEDVEG) described by Schoenfeld et al. [28] was developed and adjusted for time and instructional format. The condensed protocol was validated on a blinded academic, untrained in ultrasound, but with veterinary technology experience, and optimized in accordance with the feedback observed. Photos (control) and video (trial) were obtained during the optimization stage.

Photos were taken of the DFM on an Apple iPhone XR (San Jose, California, USA). The photos were added to the written instructions for the control group (Appendix SA). View‐by‐view video demonstration was also filmed on an Apple iPhone XR (San Jose, California, USA). The video (Appendix SB) was edited using Apple iMovie (Version 10.3.4; San Jose, California, USA) and reviewed by a small animal surgical academic.

### 2.2 Ultrasound Equipment

A Logiq E ultrasound (Logiq, New York, USA) with a 12 MHz linear transducer was used for all components of the study. Ultrasound settings as predetermined during Stage 1 simulation testing (Figure 2) were used for all students. The following ultrasound settings were determined for the simulation model and diagnostic protocol: dynamic range 75 dB, depth 2.5 cm, focus position 0.5 cm, gain 50, and transducer frequency 12 MHz.

### 2.3 Student Testing

Fourth‐year veterinary science students at Charles Sturt University (CSU) (Human ethics: H22163) were recruited to participate in the study. Participants were randomly allocated to either the video instructions (trial) group (*n* = 19) or the written instructions (control) group (*n* = 19). Within these groups, students were required to: (1) complete a preparticipation survey, (2) engage with the preprotocol instructions, (3) attempt the ultrasound examination, and (4) complete the postparticipation survey.

Three DFM were used within the study. These models were chosen based on forelimb size, VFB visibility, and location of the embedded seed. One forelimb model contained a seed in the interdigital webbing of the 3rd and 4th digits, the second contained a seed on the palmar surface of metacarpal 3 and 4, and the final forelimb model contained a seed on the dorsal surface of metacarpal 3 and 4. Students within each group (control and trial) were randomly assigned one of these three DFM using a spreadsheet formula (Microsoft Excel, Version 16.63.1).

Within the preparticipation survey, students were asked four questions. Questions were related to ultrasound use observed in clinical practice, ultrasound experience, and perceived confidence (Table S1). Additionally, as 4th year students, they were asked if they had completed their 8 h ultrasound rotation for the year.

All participants were shown reference photos of the dried wild oaten (*Avena fatua*) grass seed. Students were provided both the ultrasonographic and physical seed appearance during the preprotocol instructions (Figure 3). Participants were also instructed on how to change the depth and focal position and how to use the freeze and store button and the probe orientation notch. Transverse and sagittal orientation requirements were explained. Students were provided with ultrasound gel (Aquasonic 100 and Transonic), a sandbag, and gloves.

During the ultrasound protocol phase, student resources varied depending on their group. The control group was given instruction cards for each step, which included a still image (Appendix SA). The trial group was shown a view‐by‐view video demonstration of the protocol with additional explanation of probe techniques to enhance visualization (Appendix SB). An assessor‐to‐student ratio of 1:2 was used for each group, with the same assessor used for all participants. Students were given a scanning time limit of 4 min per sagittal view and 2 min per transverse view, as determined during model design testing phases, and were graded using a binary scale on their ability to identify the VFB.

As part of the postparticipation survey, students were asked three questions. The questions were linked to the student's ability to follow the protocol, perceived confidence upon completion of the task, and whether ultrasound use in the future was likely. Students completed the confidence survey before being advised whether they had correctly identified the VFB.

### 2.4 Statistical Analysis

A power analysis was performed to determine the minimum number of students required to participate in the project. This was conducted using internet software (ClinCalc LLC, 2022) with a significance level (alpha) of 0.05 and power of 80%.

Binary and 5‐point Likert scales were used to assess survey questions. Survey data were converted to dichotomous outcomes (yes = 1, no = 0), whereas Likert scale responses were converted to numeric values for interpretation; 1 = strongly disagree, 2 = disagree, 3 = neither agree nor disagree, 4 = agree, 5 = strongly agree. Observational data (ability to locate the VFB) were scaled as a dichotomous outcome.

Statistical analysis was performed using GraphPad Prism (GraphPad Software LLC, Version 9.5.1; Boston, USA). Observational data, specifically whether students located the grass seed, were analyzed using a two‐sided Fisher's exact test. An odds ratio and a reciprocal odds ratio were conducted. Statistical significance was determined at a level of *p* ≤ .05.

## 3 Results

### 3.1 Student Demographics

A minimum sample size of 16 participants in each group (total = 32) was required for 80% power to detect 5% variation in the parameters measured. For this study, a total of 38 participants were recruited, with 19 participants placed in the trial (written plus visual instruction) group and 19 participants placed in the control (written instructions only) group.

Participant exposure to ultrasound was common. All participants (*n* = 38) had seen ultrasound used in a veterinary practice, and 86.8% (*n* = 33) of student participants had attended CSU's 4th‐year veterinary science ultrasound practical. Interestingly, 21% of participants identified that they had seen an ultrasound used in equine practice for distal limbs, but, in comparison, only 5% (*n* = 2) of participants had seen it used on the distal limb in small animal veterinary practice.

**FIGURE 4:**
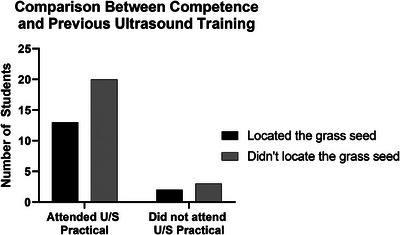
Comparison graph between attendance at previous ultrasound practical and students who located the grass seed (competent) and students who did not locate the grass seed (incompetent).

When comparing observational data (ability to locate the grass seed) and preparticipation questionnaire results, there was no subjective difference between the participants’ ability to locate the grass seed (i.e. competence) and previous ultrasound training. Only 39% of the participants (*n* = 13) who attended the ultrasound practical were able to locate the grass seed (Figure 4).

### 3.2 Vegetal Foreign Body Identification

#### 3.2.1 Teaching Modalities

**FIGURE 5:**
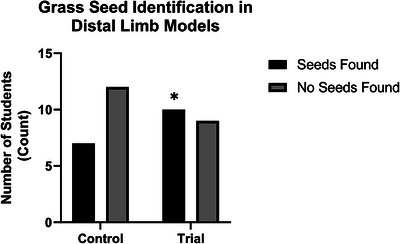
Comparison graph of students who found the seed versus students who did not find the seeds, divided by trial and control group. The level of significance is indicated by the asterisk, whereby *p < 05.

Variation in teaching methodology was observed to impact VFB‐identification success rates. Within the written protocol (control) group, 37% (*n* = 7/19) of participants successfully identified the VFB. In comparison, the written protocol and video (trial) group 53% (*n* = 10/19) of participants successfully identified the VFB. A two‐sided Fisher's exact test determined a p value of 0.0327, revealing a statistically significant (*p* < .05) difference between students identifying grass seeds in distal limb models when given a written protocol compared with a video protocol (Figure 5). Odds ratios were also analyzed, revealing an odds ratio of 0.5 and a reciprocal odds ratio of 1.92, indicating students who engaged in a videographic form of the protocol were almost twice as likely to locate the grass seed as students who were given a written protocol only.

#### 3.2.2 VFB Location

Using the canine DFM and the predetermined protocol, 45% of students (*n* = 17) were able to identify the VFB. Regardless of grouping, students who were allocated the DFM where the grass seed was placed in the interdigital web were able to identify the grass seed more frequently than other students, with 54% of participants (*n* = 7) allocated this forelimb model successfully locating the VFB. For the DFM with the grass seed on the palmar surface of the metacarpals, 50% of participants (*n* = 6) were able to identify the grass seed. Comparatively, 31% of students (*n* = 4) identified the grass seed in the DFM with the grass seed on the dorsal surface of the metacarpals.

### 3.3 Student Confidence

**FIGURE 6:**
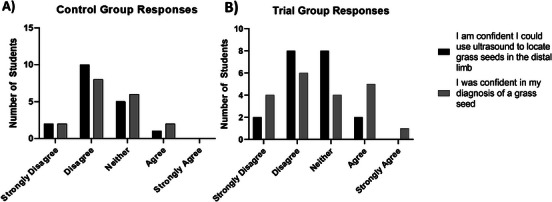
Comparison graph of confidence in the control group (A) and trial group (B) prior to performing the protocol (“I am confident I could use ultrasound to locate grass seeds in the distal limb”) and after performing the protocol (“I was confident in my diagnosis of a grass seed”).

Participants were asked a series of questions regarding their confidence in using ultrasound to locate grass seeds in the distal limb. In the control group, 63% (*n* = 12) initially disagreed or strongly disagreed with the statement *“I am confident I could use ultrasound to locate grass seeds in the distal limb”*, and 26% (*n* = 5) were neutral, while 1 participant (0.05%) agreed; no one strongly agreed (median score 2/5; IQR 2–3). Postparticipation, 52% (*n* = 10) disagreed or strongly disagreed with the statement *“I was confident in my diagnosis of a grass seed”*, 32% (*n* = 6) were neutral, and two participants (1%) agreed, again with no strong agreement (median score 2/5; IQR 2–3) (Figure 6A).

Confidence in using ultrasound to locate grass seeds in the distal limb was similar in the trial group. In the trial group, 52% (*n* = 10) initially disagreed or strongly disagreed with the statement *“I am confident I could use ultrasound to locate grass seeds in the distal limb”*, 42% (*n* = 8) were neutral, and two participants (1%) agreed, with no strong agreement (median score 2.5/5; IQR 2–3). Postparticipation results showed a wider range of confidence levels: 50% (*n* = 10) disagreed or strongly disagreed with the statement *“I was confident in my diagnosis of a grass seed”*, 21% (*n* = 4) were neutral, and 31% (*n* = 6) agreed or strongly agreed (median score 2.5/5; IQR 2–4) (Figure 6B).

**FIGURE 7:**
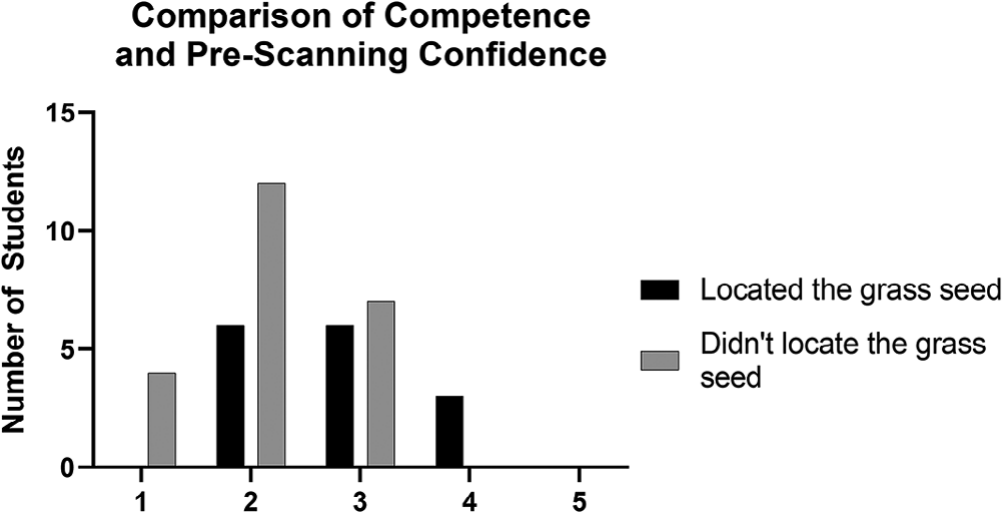
Comparison graph of student ability to locate grass seed (competence) and prescanning confidence (Likert scale responses converted to numeric values; 1 = strongly disagree, 2 = disagree, 3 = neither agree nor disagree, 4 = agree, 5 = strongly agree).

Students who identified the VFBs had higher prescanning confidence than students who did not identify the grass seed. Sixteen participants (69.5%) who did not locate the grass seed disagreed or strongly disagreed with the statement “*I am confident I could use ultrasound to locate grass seeds in the distal limb*”, whilst only 40% of participants (*n* = 6) who located the grass seed disagreed with the same statement. All participants who agreed with the statement located the grass seed (Figure 7).

**FIGURE 8:**
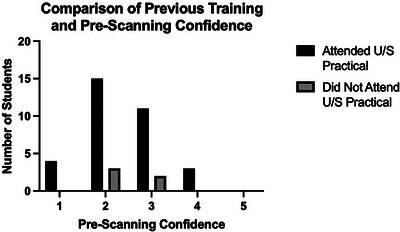
Comparison graph of prior attendance at ultrasound practical and prescanning confidence of students (Likert scale responses converted to numeric values; 1 = strongly disagree, 2 = disagree, 3 = neither agree nor disagree, 4 = agree, 5 = strongly agree).

There was no apparent effect of prior ultrasound practical attendance on prescanning confidence. Survey data revealed that only 0.09% of students (*n* = 3) who attended the ultrasound practical agreed with the statement “*I am confident I could use ultrasound to locate a vegetal foreign body in the distal limb*”, and no one strongly agreed. Figure 8 shows the graphical distribution of students’ self‐assessment of preparticipation confidence, comparing prior attendance at the ultrasound practical.

Confidence postparticipation increased confidence regardless of whether the student identified the VFB. Overall, 33.3% of participants (*n* = 5) who located the grass seed either agreed or strongly agreed with the statement “*I was confident in my diagnosis of a grass seed*”. Meanwhile, 13% of participants (*n* = 3) who did not locate the grass seed also agreed with the statement. There was a wider range of responses in the postparticipation confidence survey than in the preparticipation confidence survey.

### 3.4 Student Postparticipation Survey

Students’ ability to follow the protocol was high. Postparticipation survey results revealed that 97% of participants (*n* = 37) were able to follow the protocol in the given format, with just one participant identifying that they neither agreed nor disagreed with the statement. In addition, 100% of participants either agreed (*n* = 19) or strongly agreed (*n* = 19) that they would consider using ultrasound in practice after following the protocol. Table 1 shows postparticipation survey results delineated by VFB location. Students with the VFB located in the interdigital webbing had a larger range of confidence answers than students with the grass seed located in the dorsal or palmar metacarpals.

**TABLE 1:** Postparticipation survey results with the number of students divided by allocated limb and the location of the grass seed.

		Number of students				
Question	Grass seed location	Strongly agree (5)	Agree (4)	Neither (3)	Disagree (2)	Strongly disagree (1)
I was able to follow the protocol in the format it was given	Interdigital Web	6	7			
	Dorsal Metacarpals	2	11			
	Palmar Metacarpals	5	6	1		
I was confident in my diagnosis of a grass seed	Interdigital Web	1	3	1	5	3
	Dorsal Metacarpals		1	6	6	
	Palmar Metacarpals		3	3	3	3
I would consider using ultrasound on veterinary placement	Interdigital Web	5	8			
	Dorsal Metacarpals	5	8			
	Palmar Metacarpals	9	3			

## 4 Discussion

Simulation‐based learning is becoming readily available for teaching in veterinary medicine. Model use has been well‐documented in surgery and anesthesiology, and is a widely accepted tool used to enhance the student learning experience and improve competency‐based developmental outcomes [29, 30]. However, limited research has been conducted on the use of simulation models in ultrasonography. This study is the first to assess the use of simulation models on student outcomes in identifying VFB in canine distal limb simulation models using two different teaching modalities (video demonstration vs. written instruction). The results highlight that simulation models are a valuable tool for teaching ultrasonography and demonstrate that students benefit most when utilizing mixed modalities (video or visual aids and written instruction), rather than relying on one teaching modality when learning in a consequence‐free environment.

Simulation models have been found to improve participant skills and confidence when performing a task [29]. Simulators serve as a valuable tool for integrating alternative methods in conjunction with ethically sourced cadavers or live patients [14, 15]. Studies have indicated that veterinary students find the utilization of simulation models a beneficial tool for teaching small animal dentistry [18], as it provides students time to develop skills and confidence beforehand in a consequence‐free environment. The results from this study found a similar positive trend in both groups when comparing pre‐ and post‐ survey confidence questions. This, along with all students agreeing that they would consider using ultrasonography to identify canine distal limb VFBs, reveals that simulation models in a supportive learning environment (regardless of teaching modality) enable students to practice without fear of mistakes, fostering confidence and emotional preparedness.

The use of multiple teaching modalities can positively enhance student learning outcomes. In the past, the incorporation of multiple teaching modalities has been shown to improve knowledge retention more effectively than repetition of words alone. This is because words can be abstract and/or challenging for the brain to organize and store [23]. Within this study, a similar result was found with the use of multiple teaching modalities being more beneficial for student learning. Participants provided video and written instructions (trial group) were 1.92 times more likely to identify the VFB within the simulation model when compared with the control group. This finding illustrates that simulation models offer both tactile and visual input, and when combined with the verbal and visual elements of video demonstrations, increase students’ ability to locate the grass seed more effectively than written instructions alone. Furthermore, with just 5% of students having had previous exposure to distal limb ultrasonography in practice, this finding can be attributed solely to the varying teaching modalities.

Ultrasonography is a complex veterinary skill that requires repeated practice. Users must know how to operate the machine effectively, correctly identify common artifacts, and have detailed knowledge of the regional anatomy being examined [31]. Within this study, the results confirmed the importance of these ultrasound fundamentals. Fifty‐five percent of participants were unable to locate the grass seed due to a knowledge gap in either anatomy or the physics of ultrasonography, or both. Furthermore, no significant difference was observed in VFB identification between those who had completed (*n* = 34) 8 h of introductory ultrasound theory and practical lessons on machine basics and canine abdominal ultrasonography when compared with those who had not (*n* = 4). This finding suggests that previous introductory exposure to ultrasonography is inadequate and that repeated practice is essential for skill development. Through the development of the canine DFM accompanied by the video protocol resource, repeated exposure is possible to further enable repeated inconsequential practice and improve student learning outcomes and confidence.

A correlation was observed between confidence and competence, as all students who reported high levels of confidence before participation were able to locate the seed. Although not significant in this study due to sample size, similar studies raise the question of whether increased confidence leads to greater competence or if greater competence leads to increased confidence [24]. Future research could therefore explore this by comparing undergraduate veterinary students with veterinary professionals (1–5 years postgraduation). Such a comparison might reveal whether confidence plays a role in participant performance and how years of clinical experience relate to the perceived effectiveness of simulation models and teaching techniques.

The use of multiple teaching modalities in combination with canine distal limb models can enhance veterinary students’ undergraduate learning. Students are almost two times more likely to identify a VFB when provided with both visual and written instructions than when provided with written instructions alone. The results of this study suggest that improved knowledge retention and understanding occur when multiple teaching modalities are used. Therefore, future studies utilizing these simulation models should consider increasing participant numbers to better evaluate student confidence in a consequence‐free, self‐directed learning environment, and also assess clinical skills acquisition using objective structured clinical examinations.

In conclusion, the development of visual teaching aids to complement distal limb simulation models [22] presents promising opportunities for advancing teaching methods and potential curriculum reforms. Further research needs to be undertaken to evaluate whether the simulation model, when combined with the teaching resources, will improve student confidence and competence when participants are given the opportunity for repeated practice through the use of an objective structured clinical examination. Additionally, further work is warranted on the use of teaching modalities and clinical simulation models in the area of ultrasonography to develop programs to not only educate veterinary science students but also provide professional development resources to current practicing clinicians.

## Conflicts of Interest

The authors declare no conflicts of interest.

## Previous Presentation or Publication Disclosure

The authors declare that this research has not been presented or published anywhere else.

## Supporting information




**Supporting File**: vru70073‐sup‐0001‐SuppMat.docx

**Supporting File**: vru70073‐sup‐0002‐Appendix A.pdf

**Supporting File**: vru70073‐sup‐0003‐Appendix B.docx

**Supporting File**: vru70073‐sup‐0004‐Appendix B.mp4

## Data Availability

Readers can obtain further information and access related data supporting the results in this paper by contacting the corresponding author.

## References

[1] M. Combs , A. Decker , P. Young , et al., “Grass Seed Foreign Body‐Related Disease in Dogs and Cats: A Wide Spectrum of Clinical Presentations,” Australian Veterinary Practitioner 47 (2017): 13–24.

[2] A. Hicks , D. Golland , J. Heller , R. Malik , and M. Combs , “Epidemiological Investigation of Grass Seed Foreign Body‐Related Disease in Dogs of the Riverina District of Rural Australia,” Australian Veterinary Journal 94, no. 3 (2016): 67–75, 10.1111/avj.12414. 26914952

[3] L. Armbrust , D. Biller , M. Radlinsky , and J. Hoskinson , “Ultrasonographic Diagnosis of Foreign Bodies Associated With Chronic Draining Tracts and Abscess in Dogs,” Veterinary Radiology & Ultrasound 44, no. 1 (2003): 66–70, 10.1111/j.1740-8261.2003.tb01452.x. 12620054

[4] E. Fauchon , C. Lassaigne , G. Ragetly , and E. Gomes , “Ultrasound‐Guided Removal of Vegetal Foreign Bodies in the Lower Extremities of Dogs: A Retrospective Study of 19 Cases,” Vlaams Diergeneeskundig Tijdschrift 86, no. 5 (2017): 285–290, 10.21825/vdt.v86i5.16167.

[5] S. Manfredi , G. Covi , M. Bonazzi , et al., “Ultrasound‐Guided Removal of Soft Tissue Foreign Bodies in Companion Animals: A Case Series,” Veterinarni Medicina 65, no. 2 (2020): 49–55, 10.17221/18/2019-VETMED.

[6] K. L. Staudte , B. J. Hopper , N. R. Gibson , and R. A. Read , “Use of Ultrasonography to Facilitate Surgical Removal of Non‐Enteric Foreign Bodies in 17 Dogs,” Journal of Small Animal Practice 45, no. 8 (2006): 395–400, 10.1111/j.1748-5827.2004.tb00254.x. 15352408

[7] M. Blondel , J. Sonet , T. Cachon , E. Ségard‐Weisse , F. X. Ferrand , and C. Carozzo , “Comparison of Imaging Techniques to Detect Migrating Foreign Bodies. Relevance of Preoperative and Intraoperative Ultrasonography for Diagnosis and Surgical Removal,” Veterinary Surgery 50, no. 4 (2021): 833–842, 10.1111/vsu.13607. 33754391

[8] G. Gnudi , A. Volta , M. Bonazzi , M. Gazzola , and G. Bertoni , “Ultrasonographic Features of Grass Awn Migration in the Dog,” Veterinary Radiology & Ultrasound 46, no. 5 (2005): 423–426, 10.1111/j.1740-8261.2005.00077.x. 16250402

[9] D. Della Santa , F. Rossi , F. Carlucci , M. Vignoli , and P. Kircher , “Ultrasound‐Guided Retrieval of Plant Awns,” Veterinary Radiology & Ultrasound 49, no. 5 (2008): 484–486, 10.1111/j.1740-8261.2008.00413.x. 18833960

[10] D. Caivano , F. Corda , A. Corda , G. Moretti , and A. Bufalari , “Application of Ultrasound in Detecting and Removing Migrating Grass Awns in Dogs and Cats: A Systematic Review,” Animals 13, no. 13 (2023): 2071, 10.3390/ani13132071. 37443870 PMC10340067

[11] J. Turner , C. H. Wilde , K. C. Hughes , J. W. Meilstrup , and E. K. Manders , “Ultrasound‐Guided Retrieval of Small Foreign Objects in Subcutaneous Tissue,” Annals of Emergency Medicine 29, no. 6 (1997): 731–734, 10.1016/S0196-0644(97)70192-6. 9174516

[12] J. Wichtel , A. Zur Linden , D. Khosa , A. Singh , W. Sears , and J. Phillips , “Validation of a Novel Ultrasound Simulation Model for Teaching Foundation‐Level Ultrasonography Skills to Veterinary Students,” Journal of Veterinary Medical Education 49, no. 4 (2022): 473–483, 10.3138/jvme-2020-0123. 34076571

[13] E. M. Scallan , A. K. Voges , K. P. Chaney , C. D. Coursey , and B. T. Simon , “The Effects of Content Delivery Methods on Ultrasound Knobology and Image Quality Recognition Training in First‐Year Veterinary Students,” Journal of Veterinary Medical Education 48, no. 1 (2021): 65–70, 10.3138/jvme.2019-0014. 31738682

[14] R. J. Scalese and S. B. Issenberg , “Effective Use of Simulations for the Teaching and Acquisition of Veterinary Professional and Clinical Skills,” Journal of Veterinary Medical Education 32, no. 4 (2005): 461–467, 10.3138/jvme.32.4.461. 16421829

[15] S. Martinsen and N. Jukes , “Towards a Humane Veterinary Education,” Journal of Veterinary Medical Education 32, no. 4 (2005): 454–460, 10.3138/jvme.32.4.454. 16421828

[16] J. A. Noyes , K. J. Carbonneau , and S. M. Matthew , “Comparative Effectiveness of Training With Simulators versus Traditional Instruction in Veterinary Education: Meta‐analysis and Systematic Review,” Journal of Veterinary Medical Education 49, no. 1 (2022): 25–38, 10.3138/jvme-2020-0026. 33891532

[17] G. C. Musk , T. Collins , and G. Hosgood , “Teaching Veterinary Anesthesia: A Survey‐Based Evaluation of Two High‐Fidelity Models and Live‐Animal Experience for Undergraduate Veterinary Students,” Journal of Veterinary Medical Education 44, no. 4 (2017): 590–602, 10.3138/jvme.0216-043R1. 28657484

[18] R. H. Lumbis , S. P. Gregory , and S. Baillie , “Evaluation of a Dental Model for Training Veterinary Students,” Journal of Veterinary Medical Education 39, no. 2 (2012): 128–135, 10.3138/jvme.1011.108R. 22717999

[19] S. Baillie , R. Christopher , A. J. Catterall , et al., “Comparison of a Silicon Skin Pad and a Tea Towel as Models for Learning a Simple Interrupted Suture,” Journal of Veterinary Medical Education 47, no. 4 (2020): 516–522, 10.3138/jvme.2018-0001. 31738680

[20] D. A. F. da Silva , A. A. Fernandes , A. E. Ventrone , et al., “The Influence of Low‐Fidelity Simulator Training on Canine Peripheral Venous Puncture Procedure,” Veterinary World 14, no. 2 (2021): 410, 10.14202/vetworld.2021.410-418. 33776306 PMC7994116

[21] M. Aulmann , M. März , I. A. Burgener , M. Alef , S. Otto , and C. K. Mülling , “Development and Evaluation of Two Canine Low‐Fidelity Simulation Models,” Journal of Veterinary Medical Education 42, no. 2 (2015): 151–160, 10.3138/jvme.1114-114R. 25862399

[22] E. Schoenfeld , F. Stanley , M. Combs , E. Callcott , A. Williams , and R. Rotne , “The Construction of Canine Distal Limb Models Used in Teaching Sonography Identification of Vegetal Foreign Bodies,” Veterinary Radiology & Ultrasound 65, no. 5 (2024): 486–495, 10.1111/vru.13379. 38712878

[23] R. S. Aisami , “Learning Styles and Visual Literacy for Learning and Performance,” Procedia‐Social and Behavioral Sciences 176 (2015): 538–545, 10.1016/j.sbspro.2015.01.508.

[24] J. Clanton , A. Gardner , M. Cheung , L. Mellert , M. Evancho‐Chapman , and R. L. George , “The Relationship Between Confidence and Competence in the Development of Surgical Skills,” Journal of Surgical Education 71, no. 3 (2014): 405–412, 10.1016/j.jsurg.2013.08.009. 24797858

[25] F. Biwer , E. MGAo , P. Aalten , and A. B. H. de Bruin , “Fostering Effective Learning Strategies in Higher Education—A Mixed‐Methods Study,” Journal of Applied Research in Memory and Cognition 9, no. 2 (2020): 186–203, 10.1016/j.jarmac.2020.03.004.

[26] L. Kandler , D. W. Tscholl , M. Kolbe , B. Seifert , D. R. Spahn , and C. B. Noethiger , “Using Educational Video to Enhance Protocol Adherence for Medical Procedures,” British Journal of Anaesthesia 116, no. 5 (2016): 662–669, 10.1093/bja/aew030. 27106970

[27] R. F. Shah and R. M. Gupta , “Video Instruction Is More Effective Than Written Instruction in Improving Inhaler Technique,” Pulmonary Pharmacology & Therapeutics 46 (2017): 16–19, 10.1016/j.pupt.2017.08.005. 28797611

[28] E. Schoenfeld , M. Combs , E. Callcott , K. Jermyn , and R. Rotne , “The Development of a Systematic Ultrasound Protocol Facilitates the Visualization of Foreign Bodies Within the Canine Distal Limb,” Frontiers in Veterinary Science (2023): 10 1298072, 10.3389/fvets.2023.1298072. 38192719 PMC10773787

[29] H. R. Braid , “The Use of Simulators for Teaching Practical Clinical Skills to Veterinary Students—A Review,” Alternatives to Laboratory Animals 50, no. 3 (2022): 184–194, 10.1177/02611929221098138. 35587390

[30] T. Kondrashova and C. Coleman , “Enhancing Learning Experience Using Ultrasound Simulation in Undergraduate Medical Education: Student Perception,” Medical Science Educator 27 (2017): 489–496, 10.1007/s40670-017-0416-2.

[31] M. Serafin‐Król and A. Maliborski , “Diagnostic Errors in Musculoskeletal Ultrasound Imaging and How to Avoid Them,” Journal of Ultrasonography 17, no. 70 (2017): 188, 10.15557/JoU.2017.0028. 29075524 PMC5647614

