# Supplementary material for: Optimizing Student Outcomes: A Comparison of Two Teaching Methods for Identifying Vegetal Foreign Bodies in Canine Limbs Using Simulation Models and Ultrasound
Source: Vet Radiol Ultrasound. 2025 Aug 20;66(5):e70073. doi: 10.1111/vru.70073 (PMC12368255; doi:10.1111/vru.70073)
Supplement: Supplementary file 1 — Supporting File: vru70073‐sup‐0001‐SuppMat.docx [file VRU-66-0-s003.docx]

**Supplementary 1: Student Questionnaires**

**Table 1:** Pre-Participation Survey

| **Question 1** | |
| --- | --- |
| Have you seen ultrasound used in practice? | Yes  No |
| If yes, on the distal limb? | Yes  No |
| **Question 2** | |
| Have you attended any practical classes on ultrasound technique? | Yes  No |
| **Question 3** | |
| What have you seen ultrasound used for? Give examples of anatomy scanned and expected diagnosis |  |
| **Question 4** | |
| I am confident I could use ultrasound to locate grass seeds in the distal limb | Strongly Agree  Agree  Neither Agree nor Disagree  Disagree  Strongly Disagree |

**Table 2:** Post-Participation Survey

| **Question 1** | |
| --- | --- |
| I was able to follow the protocol in the format it was given | Strongly Agree  Agree  Neither Agree nor Disagree  Disagree  Strongly Disagree |
| **Question 2** | |
| I was confident in my diagnosis of a grass seed | Strongly Agree  Agree  Neither Agree nor Disagree  Disagree  Strongly Disagree |
| **Question 3** | |
| I would consider using ultrasound on veterinary placement | Strongly Agree  Agree  Neither Agree nor Disagree  Disagree  Strongly Disagree |
