# Supplementary material for: Optimizing Student Outcomes: A Comparison of Two Teaching Methods for Identifying Vegetal Foreign Bodies in Canine Limbs Using Simulation Models and Ultrasound
Source: Vet Radiol Ultrasound. 2025 Aug 20;66(5):e70073. doi: 10.1111/vru.70073 (PMC12368255; doi:10.1111/vru.70073)
Supplement: Supplementary file 2 — Supporting File: vru70073‐sup‐0002‐Appendix A.pdf [file VRU-66-0-s001.pdf]

Appendix A: Student Scanning Instructions

Table 1: Ultrasound scanning protocol (adapted from Schoenfeld et al[1])

Image	Instructions	Time Limit
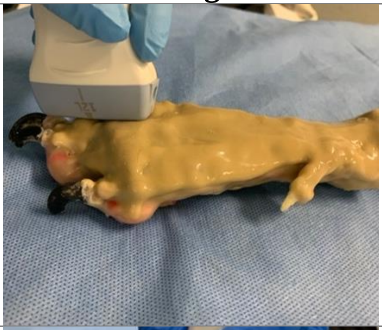	1. Place the probe on the dorsal aspect of the digits starting medially and working laterally to obtain a sagittal view	4-minutes
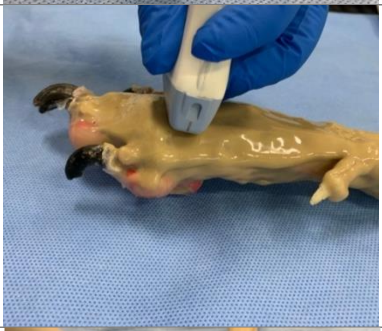	2. Move the probe to the dorsal aspect at the distal metacarpal bones viewing the junction between the head of the metacarpal bone and proximal phalanx transversely	2-minutes
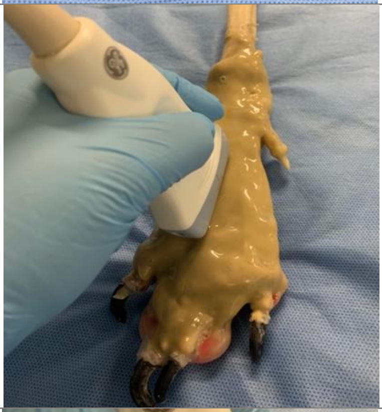	3. Turn the probe to obtain a sagittal view of the distal metacarpal bones and fan the probe as you move medially to laterally	4-minutes
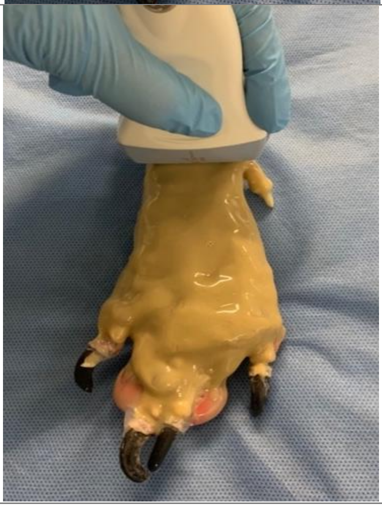	4. Move the probe to the dorsal aspect at the base of the metacarpus to obtain a transverse view; you can rock the probe back and forth to visualise between tissue layers	2-minutes

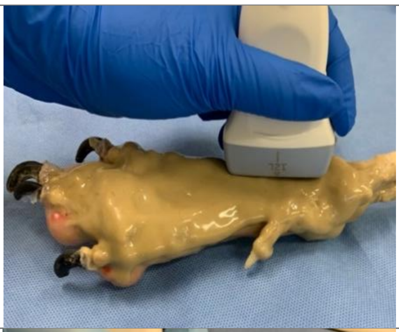	5. Turn the probe to obtain a sagittal view of the proximal aspect of the metacarpus; fan the probe moving medially to laterally	4-minutes
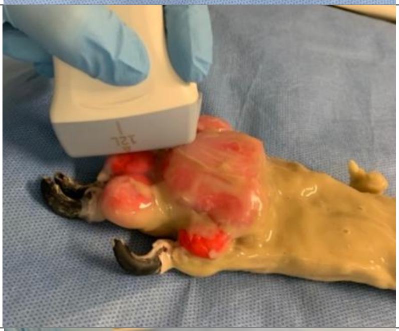	6. Turn the model over to view the palmar surface and place the probe on the palmar aspect of the digits starting medially and working laterally to obtain a sagittal view	4-minutes
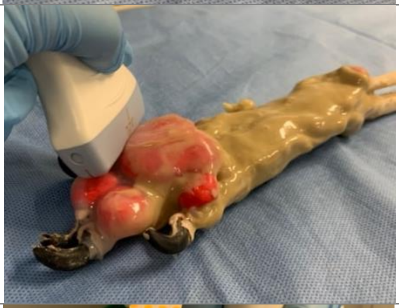	7. Place the probe on the metacarpal and digital pad triangle and obtain one view on the medial aspect and one view on the lateral aspect; in a live dog you would separate the digital and metacarpal pads and scan in between to obtain this view	2-minutes
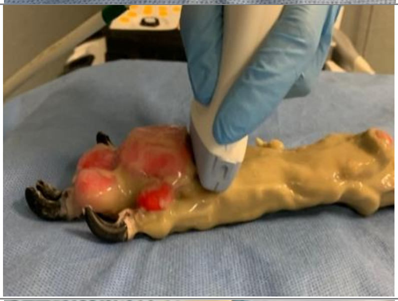	8. Move the probe to the palmar aspect just proximal to the metacarpal pad to obtain a transverse view; you can rock the probe back and forth to visualise between tissue layers	2-minutes
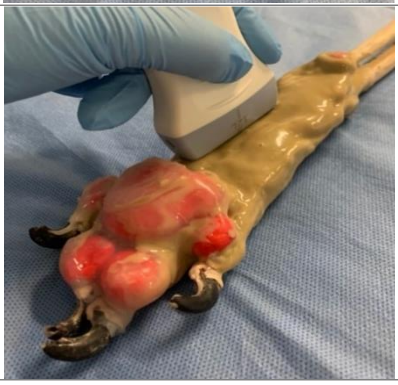	9. Turn the probe to obtain a sagittal view of the palmar aspect just proximal to the metacarpal pad at the junction of carpals and metacarpals and fan the probe moving medially to laterally	4-minutes

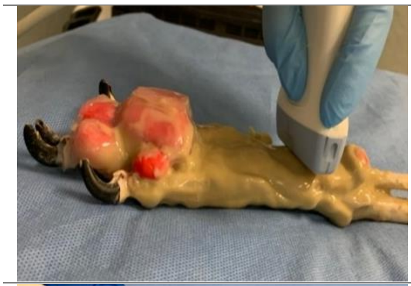	10. Move the probe to the palmar aspect just distal to the carpal pad to obtain a transverse view; rock and fan the probe to visualise between tissue layers	2-minutes
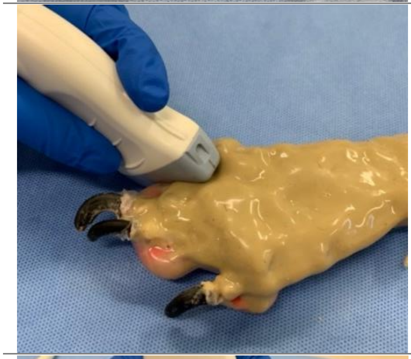	11. Place the probe on the dorsal aspect in between the digits at a 45-degree angle to scan the interdigital web and rock back and forth across the palmar and dorsal surface	4-minutes
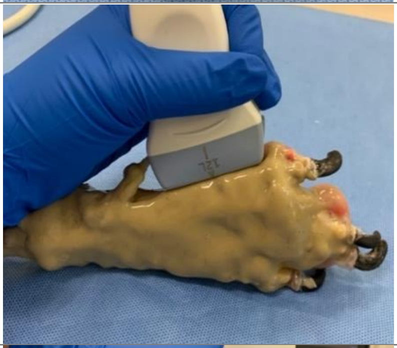	12. Place the probe on the medial surface of the limb and rock the probe side to side to change visualisation between tissue layers; if one view inadequate for full visualisation, take a second view at the proximal aspect	2-minutes
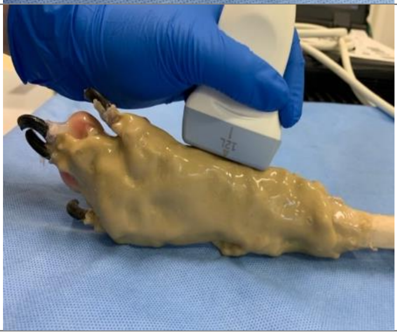	13. Move the probe to the lateral surface of the limb and rock the probe side to side to change visualisation between tissue layers; if one view inadequate for full visualisation, take a second view at the proximal aspect	2-minutes

Reference

1. Schoenfeld E, Combs M, Callcott E, Jermyn K, Rotne R. The development of a systematic ultrasound protocol facilitates the visualization of foreign bodies within the canine distal limb. *Frontiers in Veterinary Science*. 2023;10:1298072.
