# Supplementary material for: Optimizing Student Outcomes: A Comparison of Two Teaching Methods for Identifying Vegetal Foreign Bodies in Canine Limbs Using Simulation Models and Ultrasound
Source: Vet Radiol Ultrasound. 2025 Aug 20;66(5):e70073. doi: 10.1111/vru.70073 (PMC12368255; doi:10.1111/vru.70073)
Supplement: Supplementary file 3 — Supporting File: vru70073‐sup‐0003‐Appendix B.docx [file VRU-66-0-s002.docx]

[Appendix 1 - Ultrasound Protocol.mp4](https://csuprod-my.sharepoint.com/personal/eschoenfeld_csu_edu_au/Documents/Transcribed%20Files/Appendix%201%20-%20Ultrasound%20Protocol.mp4)

# [Transcript](https://csuprod-my.sharepoint.com/personal/eschoenfeld_csu_edu_au/Documents/Transcribed%20Files/Appendix%201%20-%20Ultrasound%20Protocol.mp4)

[00:00:04 – View 1](https://csuprod-my.sharepoint.com/personal/eschoenfeld_csu_edu_au/Documents/Transcribed%20Files/Appendix%201%20-%20Ultrasound%20Protocol.mp4)

[So the first view is we're going to place the probe on the dorsal aspect of the digits, starting medially and working laterally to obtain a sagittal view. So we start by placing the probe here and we can fan the probe like this to obtain visualisation between the tissue layers. And then you slowly move between each digit like this. This view has a time limit of four minutes.](https://csuprod-my.sharepoint.com/personal/eschoenfeld_csu_edu_au/Documents/Transcribed%20Files/Appendix%201%20-%20Ultrasound%20Protocol.mp4)

[00:00:33 - View 2](https://csuprod-my.sharepoint.com/personal/eschoenfeld_csu_edu_au/Documents/Transcribed%20Files/Appendix%201%20-%20Ultrasound%20Protocol.mp4)

[So we're then going to move the probe to the dorsal aspect of the distal metacarpal bones, viewing the junction between the head of the metacarpal bone and proximal phalanx transversely. So you just put it here and you will see the notch between the digits and the metacarpals, and you can again fan the probe to visualise between the tissue. This view has a time limit of two minutes.](https://csuprod-my.sharepoint.com/personal/eschoenfeld_csu_edu_au/Documents/Transcribed%20Files/Appendix%201%20-%20Ultrasound%20Protocol.mp4)

[00:00:59 - View 3](https://csuprod-my.sharepoint.com/personal/eschoenfeld_csu_edu_au/Documents/Transcribed%20Files/Appendix%201%20-%20Ultrasound%20Protocol.mp4)

[So for our next view, we turn the probe to obtain a sagittal view of the distal metacarpal bones and fan the probe as you move medially to laterally. You start here and you can fan the probe to visualise between the tissue layers and slowly move across, visualising each metacarpal bone. The time limit for this view is 4 minutes.](https://csuprod-my.sharepoint.com/personal/eschoenfeld_csu_edu_au/Documents/Transcribed%20Files/Appendix%201%20-%20Ultrasound%20Protocol.mp4)

[00:01:24 – View 4](https://csuprod-my.sharepoint.com/personal/eschoenfeld_csu_edu_au/Documents/Transcribed%20Files/Appendix%201%20-%20Ultrasound%20Protocol.mp4)

[For our next view, we are going to move the probe to the dorsal aspect at the base of the metacarpus to obtain a transverse view. So you can rock the probe back and forth again to visualise between the tissue layers. This view has a time limit of two minutes.](https://csuprod-my.sharepoint.com/personal/eschoenfeld_csu_edu_au/Documents/Transcribed%20Files/Appendix%201%20-%20Ultrasound%20Protocol.mp4)

[00:01:40 – View 5](https://csuprod-my.sharepoint.com/personal/eschoenfeld_csu_edu_au/Documents/Transcribed%20Files/Appendix%201%20-%20Ultrasound%20Protocol.mp4)

[So we are then going to move for the next view to turn the probe to obtain a sagittal view of the proximal aspect of the metacarpus. So we are going to fan the probe medially to laterally and you can rock it back and forth to obtain visualisation between the tissue layers moving across each proximal aspect of each metacarpal bone. This view has a time limit of four minutes.](https://csuprod-my.sharepoint.com/personal/eschoenfeld_csu_edu_au/Documents/Transcribed%20Files/Appendix%201%20-%20Ultrasound%20Protocol.mp4)

[00:02:07 – View 6](https://csuprod-my.sharepoint.com/personal/eschoenfeld_csu_edu_au/Documents/Transcribed%20Files/Appendix%201%20-%20Ultrasound%20Protocol.mp4)

[So we are then going to turn the model over and similar to the dorsal side, we're going to obtain a palmar surface view of the digits. So place to probe on the palmar aspect of the digits starting medially and working laterally. And you can fan the probe to increase visualisation. You can rock end to end in this view. This year has a time minute limit of four minutes.](https://csuprod-my.sharepoint.com/personal/eschoenfeld_csu_edu_au/Documents/Transcribed%20Files/Appendix%201%20-%20Ultrasound%20Protocol.mp4)

[00:02:40 – View 7](https://csuprod-my.sharepoint.com/personal/eschoenfeld_csu_edu_au/Documents/Transcribed%20Files/Appendix%201%20-%20Ultrasound%20Protocol.mp4)

[We're then going to place the probe on the metacarpal and digital pad triangle and obtain 1 view on the medial aspect and then one view on the lateral aspect. So in the live dog you'd normally separate the digital and metacarpal pads to scan between these, but we are unable to do so in this model, so we will mark this by scanning the medial and then the lateral aspect. This view has a time limit of two minutes.](https://csuprod-my.sharepoint.com/personal/eschoenfeld_csu_edu_au/Documents/Transcribed%20Files/Appendix%201%20-%20Ultrasound%20Protocol.mp4)

[00:03:12 – View 8](https://csuprod-my.sharepoint.com/personal/eschoenfeld_csu_edu_au/Documents/Transcribed%20Files/Appendix%201%20-%20Ultrasound%20Protocol.mp4)

[We're then going to move the probe to the palmar aspects just proximal to the metacarpal pad to obtain a transverse view. You can rock the probe back and forth here to visualise between the tissue layers. This view has a time limit of two minutes.](https://csuprod-my.sharepoint.com/personal/eschoenfeld_csu_edu_au/Documents/Transcribed%20Files/Appendix%201%20-%20Ultrasound%20Protocol.mp4)

[00:03:32 – View 9](https://csuprod-my.sharepoint.com/personal/eschoenfeld_csu_edu_au/Documents/Transcribed%20Files/Appendix%201%20-%20Ultrasound%20Protocol.mp4)

[For our next view, we are going to turn the probe to obtain a sagittal view of the palmar aspect, just proximal to the metacarpal pad at the junction of the carpals and metacarpals. Fan the probe moving medially to laterally looking at each metacarpal bone as you go. This view has a time limit of four minutes.](https://csuprod-my.sharepoint.com/personal/eschoenfeld_csu_edu_au/Documents/Transcribed%20Files/Appendix%201%20-%20Ultrasound%20Protocol.mp4)

[00:03:54 – View 10](https://csuprod-my.sharepoint.com/personal/eschoenfeld_csu_edu_au/Documents/Transcribed%20Files/Appendix%201%20-%20Ultrasound%20Protocol.mp4)

[Move the probe to the palmar aspect just distal to the carpal pad to obtain a transverse view. You can fan the probe and rock the probe back and forth to obtain visualisation between the tissue layers. this view has a time limit of two minutes.](https://csuprod-my.sharepoint.com/personal/eschoenfeld_csu_edu_au/Documents/Transcribed%20Files/Appendix%201%20-%20Ultrasound%20Protocol.mp4)

[00:04:14 – View 11](https://csuprod-my.sharepoint.com/personal/eschoenfeld_csu_edu_au/Documents/Transcribed%20Files/Appendix%201%20-%20Ultrasound%20Protocol.mp4)

[So then we're going to turn the probe at a 45° angle to visualise between the interdigital web. You can rock the probe back and forth between the palmar and dorsal surface to visualise between each interdigital web and again move medially to laterally.](https://csuprod-my.sharepoint.com/personal/eschoenfeld_csu_edu_au/Documents/Transcribed%20Files/Appendix%201%20-%20Ultrasound%20Protocol.mp4)

[00:04:38 – View 12](https://csuprod-my.sharepoint.com/personal/eschoenfeld_csu_edu_au/Documents/Transcribed%20Files/Appendix%201%20-%20Ultrasound%20Protocol.mp4)

[So our next view will look at the medial aspect of the limb. So we just put the probe on the medial side and you can rock the probe again to visualise between the tissue layers. If one view is not adequate for full visualisation, then you can take a second at the proximal aspect.](https://csuprod-my.sharepoint.com/personal/eschoenfeld_csu_edu_au/Documents/Transcribed%20Files/Appendix%201%20-%20Ultrasound%20Protocol.mp4)

[00:04:59 – View 13](https://csuprod-my.sharepoint.com/personal/eschoenfeld_csu_edu_au/Documents/Transcribed%20Files/Appendix%201%20-%20Ultrasound%20Protocol.mp4)

[For the final view we are going to place the probe on the lateral surface of the limb and rock the probe side to side to change visualisation between tissue layers. Similar to the medial side, if one view is inadequate for full visualisation, take a second view at the proximal aspect, repeating the rocking. This view has a time limit of two minutes.](https://csuprod-my.sharepoint.com/personal/eschoenfeld_csu_edu_au/Documents/Transcribed%20Files/Appendix%201%20-%20Ultrasound%20Protocol.mp4)
